# Differentiating Sporadic and Familial Adenomatous Polyposis-Associated Congenital Hypertrophy of the Retinal Pigment Epithelium From Congenital Grouped Pigmentation: A Review of Morphologic, Histopathologic, and Multimodal Imaging

**DOI:** 10.7759/cureus.113498

**Published:** 2026-07-28

**Authors:** Abhiram R Manda, Kiara Machado, Vikram Sivakumar, Gianluca Ramirez, Nikita K Patel, Sean Mahajan, Vasudeva R Samala, Vinitha R Samala

**Affiliations:** 1 Ophthalmology, Vanderbilt University Medical Center, Nashville, USA; 2 Ophthalmology, Augusta University, Augusta, USA; 3 Ophthalmology, University of Florida, Gainesville, USA; 4 Ophthalmology, Tanvi Eye Hospital, Hyderabad, IND

**Keywords:** colorectal cancer, congenital hypertrophy of the retinal pigment epithelium (chrpe), familial adenomatosis polyposis (fap), multimodal ophthalmic imaging, retinal pigmented epithelium, turcot syndrome

## Abstract

Congenital hypertrophy of the retinal pigment epithelium (CHRPE) occurs both as a sporadic finding and as an extraintestinal manifestation of familial adenomatous polyposis (FAP). Congenital grouped pigmentation (CGP, "bear tracks") represents a third distinct entity that may be confused with FAP-associated lesions. Accurate differentiation among these three entities has important clinical implications for appropriate surveillance, counseling, and patient management. Although prior reviews have synthesized the morphologic, histopathologic, and multimodal imaging features of pigmented retinal pigment epithelium (RPE) lesions, subsequent multimodal imaging studies and evolving evidence have refined the distinctions among FAP-associated lesions, sporadic solitary CHRPE, and CGP. This narrative review provides an updated synthesis on the clinical, fundoscopic, optical coherence tomography (OCT), fundus autofluorescence (FAF), OCT angiography (OCTA), and histopathologic features of FAP-associated CHRPE, sporadic solitary CHRPE, and CGP. Key differentiating features of FAP-associated lesions include multiplicity, bilaterality, absence of lacunae, absence of cystoid edema, and presence of pigment epithelial detachments (PEDs). CGP is distinguished by its wedge-shaped (sectorial) distribution pattern, preserved outer retinal architecture on OCT, and retention of normal ellipsoidal pigment granule morphology on histopathology. An evidence-based diagnostic feature comparison framework is proposed to assist clinicians in determining which patients with pigmented RPE lesions warrant genetic evaluation for FAP.

## Introduction and background

Pigmented lesions at the level of the retinal pigment epithelium (RPE) are commonly encountered in clinical practice, with a prevalence of approximately 1.2% in the optometric population [1]. The differential diagnosis of flat, pigmented RPE lesions includes solitary congenital hypertrophy of the retinal pigment epithelium (CHRPE), congenital grouped pigmentation (CGP, colloquially termed "bear tracks"), familial adenomatous polyposis (FAP)-associated RPE hamartomas, torpedo maculopathy, choroidal nevus, melanocytoma, combined hamartoma of the retina and RPE, RPE adenoma, and pattern dystrophy of the RPE [2-6]. 

CHRPE is also one of the most common pseudomelanomas, accounting for approximately 6% of suspected posterior uveal melanomas in a large Western series and 12.4% in a Korean study cohort [7,8]. In a large classification of RPE tumors in 926 patients, Shields et al. found solitary CHRPE comprised 79% of all RPE tumors, followed by multifocal CHRPE (4%), and FAP-associated hamartomas (1%) [5]. It is clinically important to accurately recognize CHRPE for two reasons: to distinguish it from ocular tumors that may mimic melanoma and to differentiate sporadic CHRPE and CGP from FAP-associated lesions. 

Among these, the distinction between FAP-associated CHRPE, sporadic solitary CHRPE, and CGP carries the greatest clinical consequence. FAP-associated CHRPE is the earliest and most common extraintestinal manifestation of FAP, an autosomal dominant syndrome that carries a near-100% lifetime risk of colorectal cancer if untreated. It is present in approximately 76%-80% of FAP patients [9-11]. Bonnet et al. suggest FAP-associated CHRPE as a phenotypical marker for FAP (sensitivity ~79%, specificity ~89%), with particular attention to high-risk features like bilaterality and multiple lesions, which improve the specificity of CHRPE as a marker for FAP [9]. Ocular examination for CHRPE in combination with endoscopy and genetic testing for FAP can support earlier recognition, genetic counseling, and further diagnostic evaluation [9,10,12]. Misidentification of a sporadic lesion as FAP-associated, or, more dangerously, failure to recognize FAP-associated lesions, can lead to unnecessary testing or missed cancer diagnoses, respectively. 

This review builds on several prior syntheses. Ly et al. proposed a chair-side flowchart for differentiating pigmented RPE lesions, but it relied primarily on funduscopic characteristics, as multimodal imaging data for RPE-associated CHRPE and CGP were largely uncharacterized at the time [4]. Bonnet et al. later established CHRPE's performance as a screening marker for FAP but focused on clinical detection rather than imaging-based or histopathologic differentiation [9]. Incorporating evidence published since, the present review refines the differentiating features across all three entities and proposes a stepwise diagnostic framework integrating clinical, fundoscopic, and multimodal imaging criteria to distinguish FAP-associated CHRPE from sporadic CHRPE and CGP.

## Review

Methods 

This narrative review was conducted to synthesize the current evidence on the morphologic, histopathologic, and multimodal imaging features that distinguish FAP-associated CHRPE from sporadic solitary CHRPE and CGP. A literature search was performed using PubMed to identify relevant articles from database inception through July 2026 (final search date: July 1, 2026). 

The following search string was used: ("Retinal Pigment Epithelium"[MeSH] OR "congenital hypertrophy of the retinal pigment epithelium"[tiab] OR CHRPE[tiab] OR "RPE hamartoma"[tiab] OR "grouped pigmentation"[tiab] OR "bear track*"[tiab] OR "congenital grouped pigmentation"[tiab]) AND ("Adenomatous Polyposis Coli"[MeSH] OR "familial adenomatous polyposis"[tiab] OR FAP[tiab] OR "Gardner Syndrome"[MeSH] OR "Gardner syndrome"[tiab] OR APC[tiab] OR sporadic[tiab] OR solitary[tiab]). Observational studies, case series, systematic reviews, and histopathologic and multimodal imaging studies that characterized the clinical, fundoscopic, optical coherence tomography (OCT), fundus autofluorescence (FAF), OCT angiography (OCTA), or histopathologic features of these lesions were included, with priority given to studies that directly characterized these lesions; editorials and letters without original data were excluded. To limit selection bias, titles and abstracts were screened against these predefined eligibility criteria, and full texts of potentially eligible records were reviewed before final inclusion; disagreements regarding eligibility were resolved by discussion among the authors. Additional relevant articles were identified through manual screening of reference lists. Given the narrative nature of this review, study selection was not conducted according to a predefined protocol or Preferred Reporting Items for Systematic Reviews and Meta-Analyses (PRISMA) guidelines, and no formal risk-of-bias assessment was performed. 

Historical context 

Buettner described CHRPE as a benign, congenital lesion of the RPE, typically presenting as a solitary, flat, and well-demarcated pigmented lesion [13]. The association between pigmented RPE lesions and FAP was first described in the 1980s, when multiple, bilateral CHRPE lesions were recognized as a phenotypic marker for polyposis [12,14,15]. Shields et al. subsequently demonstrated that solitary CHRPE and CGP are not associated with FAP or Gardner syndrome, establishing that these are clinically distinct from the multiple bilateral lesions seen in polyposis patients [16]. This clinical separation was later reinforced by genotype-phenotype studies showing that pigmented fundus lesions in FAP cluster with specific adenomatous polyposis coli (APC) mutation regions. Thus, the APC mutation location may explain why FAP-associated lesions occur in selective kindreds.

Genotype-phenotype correlations 

The expression of CHRPE in FAP is strongly correlated with the position of the germline APC mutation. CHRPE is associated with mutations between codons 311 and 1444 (exon 9 through exon 15) and is systematically absent with mutations 5' of exon 9 or 3' of codon 1444 [17-19]. Valanzano et al. found that 100% of patients with exon 15 mutations (codons 876-1324) had a positive CHRPE score, compared with only 16.6% of those with exon 9 mutations [20]. Bertario et al., in a series of 953 FAP patients from 187 families, confirmed that mutations spanning codons 543-1309, though variable, were strongly associated with CHRPE [21]. Giardiello et al. similarly localized the association to codons 541-1309 [22]. Notably, CHRPE is unusual in attenuated FAP (AFAP), which is associated with mutations at the 5' end, distal 3' end, or exon 9 of the APC gene [18,23]. CHRPE is present in approximately two-thirds of FAP kindreds and absent from the remaining third; within CHRPE-positive kindreds, the retinal phenotypic expression is homogeneous [15]. 

Advances in multimodal imaging 

Early descriptions of CHRPE, FAP-associated lesions, and CGP relied largely on indirect ophthalmoscopy and fundus photography to emphasize visible features like lesion number, laterality, distribution, borders, lacunae, and halos [13,15,16,24]. More recently, multimodal imaging, including enhanced-depth imaging OCT (EDI-OCT), FAF, and OCTA, has expanded this framework to correlate morphology with underlying RPE, retinal, and vascular features [4,25-28,29]. Ly et al. provided an early synthesis of the available advanced imaging features of pigmented RPE lesions, but for FAP-associated lesions and CGP, findings from several modalities, like FAF, infrared reflectance (IR) imaging, and B-scan ultrasonography, were more limited or not yet systematically characterized [4]. 

Subsequent multimodal imaging studies have refined the distinction between CHRPE, FAP-associated lesions, and CGP, specifically targeting unique FAP-associated lesion findings [28]. Kong et al. provided a large-scale multimodal imaging characterization of FAP-associated lesions (which they term "retinal pigment epithelial hamartomas associated with FAP" or RPEH-FAP), identifying key differentiating features from sporadic CHRPE, including the absence of lacunae and cystoid edema and the presence of pigment epithelial detachments [28]. For consistency, these lesions are referred to as FAP-associated CHRPE throughout this review, with the term RPEH-FAP retained only where it reflects the original authors' nomenclature. 

Sporadic solitary CHRPE 

Sporadic solitary CHRPE presents as a single, well-circumscribed, flat, darkly pigmented lesion with a marginal halo typically located in the retinal periphery [6]. Shields et al., in a cohort of 330 patients, reported the largest median basal diameter as 4.5 mm, with lacunae present in 43% of pigmented CHRPE lesions, a surrounding pigmented halo in 57% of patients, and a surrounding nonpigmented halo in 46% of patients [6]. Fung et al. characterized 18 sporadic CHRPE lesions with EDI-OCT, finding thickened RPE (89%), irregular RPE (83%), absent RPE within lacunae (89% of imaged lacunae), outer nuclear layer thinning or absence (67%), complete photoreceptor loss (100%), cystoid edema (28%), and subretinal clefts (33%) [25]. On FAF, all lesions were hypoautofluorescent, consistent with the histopathologic absence of lipofuscin; of lesions with lacunae, isoautofluorescence (71%) and hypoautofluorescence (29%) were also seen [25,27]. On IR imaging, 94% of CHRPE lesions were hyporeflective, and 100% of imaged lacunae were hyperreflective, providing a complementary non-invasive modality for lacunae detection [25]. By ultrasonography, sporadic CHRPE displayed a mean thickness of 1.0 mm (range 0.9-1.4 mm) and a flat, acoustically solid appearance on B-scan [25]. Cohen et al. found retinal vascular changes in 91% of sporadic CHRPE lesions [30]. Among these, all showed capillary rarefaction, and one-fourth had areas of capillary nonperfusion exceeding 1 disc diameter along with microaneurysmal capillary dilatations on fluorescein angiography (FA) [30]. Sporadic CHRPE demonstrates slow flat enlargement in 83% of cases over >3 years (median 10 μm/month), and rare nodular transformation (~1% at 10 years) [5,6]. These prevalence estimates derive largely from single-center cohorts, and racial or ethnic homogeneity within these populations may limit generalizability of specific frequency figures, such as halo prevalence, to more diverse patient populations. 

FAP-associated CHRPE (RPEH-FAP) 

FAP-associated CHRPE lesions are multiple (mean ~10 per eye; range 1-31) and bilateral in 77-100% of cases [9,15,28,31]. Kong et al. identified 233 RPEH-FAP lesions in 24 eyes of 12 patients [28]. The most common morphology was small, circular pigmented dots (42.9%), predominantly located outside the posterior pole (92.7%), with the superotemporal quadrant most commonly involved (36.1%) [28]. Depigmented margins ("fish-tails") were present in 48.5% of lesions [28]. On FAF, the pigmented portion was hypoautofluorescent in 93.3%, and depigmented halos/fish-tails were isoautofluorescent (46.8%) or hyperautofluorescent (35.1%) [28]. On OCT, the RPE was thickened in 67.3%, with outer nuclear layer thinning/absence in 83.7% and ellipsoid zone absence in 93.9% [28]. Critically, no lesion demonstrated lacunae, cystoid edema, or subretinal fluid [28]. PEDs were present in 22.4%, a novel finding not previously described in any CHRPE subtype [28]. On OCTA, no vascular signal was observed in any FAP-associated lesion [28]. This finding mirrors sporadic CHRPE, in which retinal and choroidal vasculature also appear normal on OCTA, though segmentation artifacts from outer retinal thinning can interfere with interpretation [29]. However, on FA, FAP-associated lesions demonstrate normal retinal vasculature overlying the lesions with blockage of background choroidal fluorescence, in contrast to the capillary rarefaction and nonperfusion seen in sporadic CHRPE [30,32]. Because much of the detailed multimodal-imaging characterization is derived from a single series, these frequency figures should be interpreted as preliminary, pending confirmation in larger, genetically defined cohorts. 

Congenital grouped pigmentation (bear tracks) 

CGP presents as clusters of multiple small, well-demarcated, flat pigmented spots arranged in a sectorial or grouped pattern resembling animal footprints [4,24,33,34]. Each group contains 3-30 foci, with lesions often increasing in size from the optic disc toward the periphery, a distribution that has been proposed to follow developmental patterns of pigmentary mosaicism analogous to cutaneous lines of Blaschko [33]. CGP is typically unilateral and confined to one sector of the fundus, although bilateral, familial, and diffuse presentations have been reported [33-35]. On multimodal imaging, bear track lesions show uniform hypoautofluorescence on FAF and, critically, normal retinal lamination without outer retinal atrophy on SD-OCT, a key distinction from both sporadic and FAP-associated CHRPE [4,34]. Additionally, pigmented CGP lesions may not present with lacunae-associated RPE loss, cystoid change, or PEDs described in other CHRPE subtypes [4,34]. Unique CGP IR imaging, OCTA, and FA features remain limited compared with sporadic CHRPE and FAP-associated lesions [4]. 

Histopathologic distinctions 

Histopathologically, sporadic CHRPE is composed of tall, maximally pigmented RPE cells with a density 1.7x greater than adjacent normal RPE, containing macromelanosomes, abnormally large, round pigment granules resulting from disturbed melanogenesis [13,36,37]. Ultraviolet fluorescence microscopy shows no autofluorescent lipofuscin granules, suggesting that CHRPE cells lack the capacity to phagocytose photoreceptor outer segments, which may explain the overlying photoreceptor degeneration [36]. CGP differs ultrastructurally. While involved RPE cells contain increased pigment granules, the granules retain normal ellipsoidal shape, and significant hypertrophy and hyperplasia are not features found on microscopy [4,24]. Histopathologic data for FAP-associated lesions are extremely limited, since these lesions are rarely biopsied. The available evidence suggests that these lesions show hypertrophied RPE with macromelanosomes, similar to sporadic CHRPE [38]. 

Comparative feature summary 

The key morphologic, imaging, and histopathologic features distinguishing FAP-associated CHRPE, sporadic solitary CHRPE, and CGP are shown in Table 1. 

**Table 1:** Distinguishing features of FAP-associated CHRPE, sporadic solitary CHRPE, and CGP Comparative morphologic, imaging, and histopathologic features of FAP-associated CHRPE, sporadic solitary CHRPE, and CGP (bear tracks). Asterisks (*) mark the five features most strongly supported by the evidence as distinguishing FAP-associated CHRPE from sporadic CHRPE and CGP: number, laterality, lacunae, cystoid edema, and PED. FAP-associated imaging data are derived principally from a single multimodal-imaging series [28]; cystoid edema and PED figures are based on small lesion counts and warrant cautious interpretation. CGP, congenital grouped pigmentation; CHRPE, congenital hypertrophy of the retinal pigment epithelium; FAF, fundus autofluorescence; FAP, familial adenomatous polyposis; OCT, optical coherence tomography; ONL, outer nuclear layer; PED, pigment epithelial detachment.

Feature	FAP-associated CHRPE	Sporadic solitary CHRPE	Bear tracks (CGP)
* Number	Multiple (~10/eye)	Solitary	Multiple (3-30)
* Laterality	Bilateral 77-100%	Unilateral	Unilateral, sectorial
* Lacunae	Absent	Present 43-78%	Absent
* Cystoid edema	Absent	Present 28%	Not reported
* PED	Present 22.4%	Not reported	Not reported
Size	Small dots	Larger, 4.5 mm	Smaller near disc
Shape	Round / pisciform	Round / oval	Falciform, nested
Location	Outside posterior pole	Peripheral	Sectorial from disc
Halo	Fish-tail ~49%	Pigmented 78%	Not typical
OCT outer retina	ONL thinning	ONL thinning	Normal lamination
FAF (pigmented)	Hypo 93%	Hypo 100%	Hypo
Pigment granule	Macromelanosome	Macromelanosome	Normal ellipsoidal
FAP association	76-80% of FAP	None	None

Based on the synthesized evidence, several features reliably distinguish FAP-associated CHRPE from sporadic solitary CHRPE and CGP (Table 1). The most robust discriminators are lesion number and laterality: FAP-associated lesions are characteristically multiple and bilateral in 77%-100% of cases, whereas sporadic CHRPE is solitary and unilateral, and CGP is typically unilateral with a sectorial distribution [9,14-16,24,28,31,33]. Imaging-based features further sharpen this distinction. Lacunae, present in 43%-78% of sporadic CHRPE lesions, were absent in all 233 FAP-associated lesions characterized by Kong et al. and are similarly absent in CGP; their presence therefore strongly favors sporadic CHRPE and argues against FAP association [25,28]. Cystoid edema on OCT follows a comparable pattern, occurring in 28% of sporadic CHRPE but in none of the FAP-associated lesions studied, representing a novel OCT-based differentiator [3,25,28]. Conversely, pigment epithelial detachments, found in 22.4% of FAP-associated lesions, have not been described in sporadic CHRPE or CGP and represent a newly identified feature specific to FAP-associated disease [28]. FAP-associated lesions tend to present as small circular pigmented dots, in contrast to the larger median basal diameter (4.5 mm) typical of sporadic CHRPE lesions [6,25,28]. 

CGP lesions follow a distinctive sectorial distribution radiating from the optic disc, with smaller foci near the disc enlarging toward the periphery, a pattern not observed in FAP-associated or sporadic CHRPE [4,24,33-35]. Histopathologically, CGP differs from both CHRPE types in that its pigment granules retain a normal ellipsoidal shape without the marked RPE hypertrophy or hyperplasia seen in sporadic and FAP-associated CHRPE, both of which demonstrate macromelanosome formation [4,13,24,36,37]. 

Several features, by contrast, are shared across all three entities and therefore cannot be used for differentiation, including hypoautofluorescence of the pigmented portion on FAF, outer retinal thinning on OCT (present in both FAP-associated and sporadic CHRPE, though absent in CGP), and normal sublesional choroidal thickness [26,28]. 

Differentiation from choroidal melanoma 

Because CHRPE is among the most common pseudomelanomas, distinguishing it from choroidal melanoma is an important clinical consideration [7,8]. Multimodal imaging helps localize the lesion to the RPE and distinguish it from a true choroidal mass [25,28]. On FAF, CHRPE is uniformly hypoautofluorescent, whereas choroidal melanoma demonstrates diffuse or patchy hyperautofluorescence from overlying lipofuscin accumulation [25,39]. On OCT, CHRPE appears flat with thickened RPE, while melanoma shows dome-shaped elevation with “shaggy photoreceptors,” subretinal fluid, and intraretinal fluid [25,39]. On ultrasonography, CHRPE shows high internal reflectivity on A-scan with an acoustically solid flat lesion on B-scan, in contrast to melanoma’s low internal reflectivity and acoustic hollowness [3,25,40]. Additionally, the “shadow sign”-an apparent elevation with a subretinal fluid-like appearance-may be observed in CHRPE of young myopic pigmented patients due to “dark without pressure” and should not be mistaken for true subretinal fluid or lesion thickness seen in melanoma [41]. Despite these differentiating imaging features, multimodal imaging should not be used as a stand-alone diagnostic algorithm: atypical or amelanotic presentations of pigmented RPE lesions may still closely mimic choroidal melanoma, and findings should be interpreted alongside specialist ocular oncology input when the diagnosis is uncertain. 

Proposed diagnostic framework 

**Figure 1:**
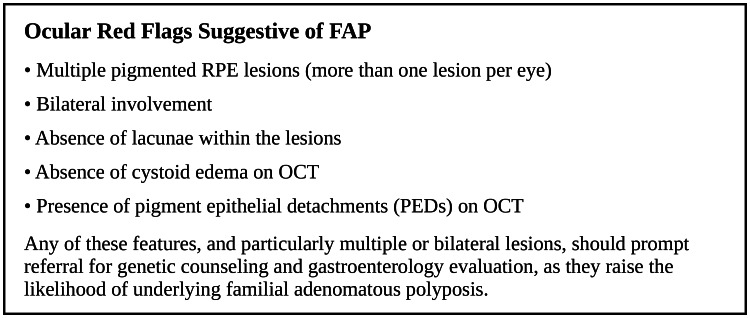
Summary of ocular red flags for FAP Ocular findings that should raise suspicion for FAP in a patient with pigmented RPE lesions and prompt referral for genetic counseling and gastroenterology evaluation. CHRPE, congenital hypertrophy of the RPE; FAP, familial adenomatous polyposis; OCT, optical coherence tomography; RPE, retinal pigment epithelium.

Based on the synthesized evidence, a stepwise approach to evaluating pigmented RPE lesions for FAP association is proposed in Table 2, and the ocular findings that should prompt referral for FAP evaluation are summarized in Figure 1. 

**Table 2:** Proposed diagnostic framework for differentiating FAP-associated CHRPE from sporadic CHRPE and CGP Proposed stepwise approach to evaluating a pigmented RPE lesion for FAP association, progressing from clinical assessment to multimodal imaging. CGP, congenital grouped pigmentation; CHRPE, congenital hypertrophy of the retinal pigment epithelium; FAP, familial adenomatous polyposis; PED, pigment epithelial detachment; RPE, retinal pigment epithelium.

Step	Assessment	Interpretation
Step 1	Number & laterality	Multiple lesions (sensitivity ~86.6%, specificity ~100%) and bilateral lesions (sensitivity ~84.3%, specificity ~100%) strongly suggest FAP [42]. A solitary unilateral lesion is most consistent with sporadic CHRPE. A unilateral sectorial cluster radiating from the optic disc suggests CGP [33].
Step 2	Fundoscopic morphology	Lacunae strongly favor sporadic CHRPE and argue against FAP [25,28]. A depigmented halo (48.5%) is common in FAP-associated lesions, with the majority containing a fish-tail pattern directed at the optic disc [28]. Nested falciform spots, or “bear tracks”, fitting into one another, are pathognomonic for CGP [33].
Step 3	Multimodal imaging	Absence of cystoid edema and presence of PEDs favor FAP-associated CHRPE [28]. Cystoid edema or subretinal clefts favor sporadic CHRPE [25]. Normal retinal lamination without outer-retinal atrophy distinguishes CGP from both CHRPE types [34].

Nomenclature considerations 

Kong et al. proposed the term "retinal pigment epithelial hamartoma associated with FAP" to distinguish these lesions from sporadic CHRPE, arguing that the term "CHRPE" has been applied too broadly to morphologically distinct entities [28]. Shields et al., in their classification of 948 RPE tumors, similarly separated "RPE hamartomas associated with FAP" from "solitary CHRPE" and "multifocal CHRPE" as distinct diagnostic categories [5]. This nomenclature shift reflects growing recognition that FAP-associated lesions are biologically and morphologically distinct from sporadic CHRPE, despite sharing some imaging features. 

Limitations of the current evidence 

Several limitations should be acknowledged. First, the majority of studies on FAP-associated CHRPE used indirect ophthalmoscopy without multimodal imaging, limiting the granularity of morphologic characterization. Few studies have systematically applied multimodal imaging to FAP-associated lesions [4,28]. Second, histopathologic data are extremely limited for FAP-associated lesions, as these are not biopsied; most histopathologic knowledge derives from sporadic CHRPE found incidentally in enucleated or autopsied eyes [24,36,37]. Third, no universally accepted scoring system or diagnostic threshold exists for CHRPE in FAP, and studies vary in their criteria, using ≥3 or ≥4 lesions, or bilaterality alone [9,14,15,42]. Fourth, the evidence base is uneven in strength: the discriminators of number and laterality derive from multiple independent cohorts, whereas the newer OCT-based features rest on comparatively few lesions from single-center series and warrant confirmation in larger, genetically confirmed populations

Future directions 

Prospective studies using standardized multimodal imaging protocols in genetically confirmed FAP cohorts are needed to validate the differentiating features identified in this review. The development of a validated scoring system incorporating both clinical and imaging features would improve diagnostic consistency. Additionally, the role of artificial intelligence in automated detection and classification of pigmented RPE lesions on widefield imaging warrants investigation. 

## Conclusions

FAP-associated CHRPE, sporadic solitary CHRPE, and CGP are three distinct entities with overlapping but distinguishable morphologic and imaging features. The key differentiating features of FAP-associated lesions are multiplicity, bilaterality, absence of lacunae, absence of cystoid edema, and presence of pigment epithelial detachments. CGP is distinguished by its sectorial distribution pattern, preserved outer retinal architecture on OCT, and retention of normal ellipsoidal pigment granule morphology. This evidence-based diagnostic framework can assist clinicians in determining which patients with pigmented RPE lesions warrant genetic evaluation for FAP. 
